# Impact of polyploidy on plant tolerance to abiotic and biotic stresses

**DOI:** 10.3389/fpls.2022.869423

**Published:** 2022-08-22

**Authors:** Vanesa E. Tossi, Leandro J. Martínez Tosar, Leandro E. Laino, Jesica Iannicelli, José Javier Regalado, Alejandro Salvio Escandón, Irene Baroli, Humberto Fabio Causin, Sandra Irene Pitta-Álvarez

**Affiliations:** ^1^Laboratorio de Cultivo Experimental de Plantas y Microalgas, Departamento de Biodiversidad y Biología Experimental (DBBE), Facultad de Ciencias Exactas y Naturales, Universidad de Buenos Aires, Ciudad Universitaria, Int. Güiraldes y Cantilo, Buenos Aires, Argentina; ^2^Facultad de Ciencias Exactas y Naturales, Consejo Nacional de Investigaciones Científicas y Tecnológicas (CONICET), Universidad de Buenos Aires, Instituto de Micología y Botánica (INMIBO), Ciudad Universitaria, Int. Güiraldes y Cantilo, Buenos Aires, Argentina; ^3^Departamento de Biotecnología, Alimentos, Agro y Ambiental (DEBAL), Facultad de Ingeniería y Ciencias Exactas, Universidad Argentina de la Empresa (UADE), Buenos Aires, Argentina; ^4^Instituto Nacional de Tecnología, Agropecuaria (INTA), Instituto de Genética “Ewald A. Favret”, Buenos Aires, Argentina; ^5^Facultad de Ciencias Exactas y Naturales, Consejo Nacional de Investigaciones Científicas y Tecnológicas (CONICET), Universidad de Buenos Aires, Instituto de Biodiversidad y Biología Experimental (IBBEA), Ciudad Universitaria, Int. Güiraldes y Cantilo, Buenos Aires, Argentina; ^6^Departamento de Biodiversidad y Biología Experimental (DBBE), Facultad de Ciencias Exactas y Naturales, Universidad de Buenos Aires, Ciudad Universitaria, Int. Güiraldes y Cantilo, Buenos Aires, Argentina

**Keywords:** abiotic stress, biotic stress, polyploids, whole genome duplication, stress tolerance, plant breeding

## Abstract

Polyploidy, defined as the coexistence of three or more complete sets of chromosomes in an organism’s cells, is considered as a pivotal moving force in the evolutionary history of vascular plants and has played a major role in the domestication of several crops. In the last decades, improved cultivars of economically important species have been developed artificially by inducing autopolyploidy with chemical agents. Studies on diverse species have shown that the anatomical and physiological changes generated by either natural or artificial polyploidization can increase tolerance to abiotic and biotic stresses as well as disease resistance, which may positively impact on plant growth and net production. The aim of this work is to review the current literature regarding the link between plant ploidy level and tolerance to abiotic and biotic stressors, with an emphasis on the physiological and molecular mechanisms responsible for these effects, as well as their impact on the growth and development of both natural and artificially generated polyploids, during exposure to adverse environmental conditions. We focused on the analysis of those types of stressors in which more progress has been made in the knowledge of the putative morpho-physiological and/or molecular mechanisms involved, revealing both the factors in common, as well as those that need to be addressed in future research.

## Introduction

Most plant lineages have undergone whole genome duplication (WGD) events in their past (Bowers et al., 2003; Paterson et al., 2004; Pfeil et al., 2005; Burleigh et al., 2008; Van de Peer et al., 2009; Shi et al., 2010; Ruprecht et al., 2017), with some lineages experiencing repeated doubling events. The fact that polyploidy shows such a remarkable high frequency of occurrence among plant species is compelling from an adaptive point of view. Comparative distributions of gene duplication times and orthologue divergence times suggest 244 ancient WGD events across *Viridiplantae* (Leebens-Mack et al., 2019). The expansions (along with subsequent possible contractions; Simonin and Roddy, 2018) in gene families after WGDs contributed to the dynamic evolution of metabolic, regulatory and signaling networks in polyploids (Maere et al., 2005; Hanada et al., 2008). This plasticity may represent opportunities for adaptation, especially under fluctuations in the selective pressure exerted by the environment, or it may simply provide significant variation for neofunctionalization and the evolution of new species (Schranz et al., 2012). But apart from this alleged role in adaptation and/or speciation in natural conditions, humanity has managed to harness the adaptive potential of genome duplications and applied it to the enhancement of stress resistance and crop production in domestic environments (Renny-Byfield and Wendel, 2014).

The evidence suggesting that environmental robustness is enhanced after WGDs (e.g., Ramsey, 2011; Madlung, 2013; Diallo et al., 2016) is supported by the fact that many WGD events can be historically correlated with periods of dramatic environmental change, usually leading to mass extinctions (Fawcett et al., 2009; Vanneste et al., 2014a,b; Cannon et al., 2015; Huang et al., 2016; Lohaus and Van de Peer, 2016; Yu et al., 2017). For example, the Cretaceous-Paleogene boundary (66 Mya) is linked to several cataclysmic events causing major climatic changes that ultimately led to the loss of roughly two thirds of animal and plant biodiversity (Petersen et al., 2016). Interestingly, this era gave rise to a wave of WGD events that affected most taxa in plants (mainly in angiosperms, and more limitedly in gymnosperms and non-seed vascular plants; Mable et al., 2011). Retention of gene duplicates after WGDs in this period appears to correlate with functions linked to adaptation to low temperatures and darkness, two environmental factors believed to be of great influence at the time (Wu et al., 2020b). It has been suggested that at least a fraction of this apparent enrichment in WGDs taking place around certain cataclysmic global events might have actually preceded the environmental turmoil by millions of years (Robertson et al., 2017; Van de Peer et al., 2021 and references therein). However, the multiplicity of dating correlations between WGDs and events of worldwide dramatic environmental change strongly supports the notion of a causative link between the two, i.e., that polyploidization might notably enhance plant fitness in the midst of harsh stressful conditions.

Provokingly, the adaptive potential derived from the plasticity shown by natural polyploids in the face of the stress imposed by environmental changes can also be regarded as a two-way phenomenon, in which the environmental stress can also facilitate the occurrence of WGD events. The production of unreduced gametes is the main mechanism that gives rise to polyploidy in nature (Chen and Ni, 2006; Soltis and Soltis, 2009). Interestingly, the frequency of occurrence of unreduced gametes has been observed to be significantly increased by changes in environmental stressors such as temperature (Mason et al., 2011; Pecrix et al., 2011; De Storme et al., 2012), nutrient shortage and leaf wounding (e.g., Sora et al., 2016), and virus-induced disease (Kostoff, 1933). Though these observations remain to be validated for other types of stress, this adds plausibility to a causative effect of general environmental changes on the frequency of WGD events.

Polyploids can be classified as autopolyploids or allopolyploids, and these can be either natural or artificial (chemically-induced; Meru, 2012; Hegarty et al., 2013). Autopolyploids result from the doubling of one chromosome set within one species; whereas in allopolyploids, chromosome sets of different species can combine through hybridization events and, subsequently, increase their number through duplication events. During polyploidization, structural and functional changes in genomes such as chromosomal rearrangements (Osborn et al., 2003; Chen et al., 2007; Xie et al., 2010; Xiong et al., 2011; Wang et al., 2012), gene loss (Buggs et al., 2009, 2012; Emery et al., 2018), epigenetic reprogramming (i.e., DNA methylation and histone modifications; e.g., Jackson and Chen, 2010; Sehrish et al., 2014; Yoo et al., 2014; Zhou et al., 2021), and miRNAome alterations (Liu and Sun, 2019), can have a huge impact on gene expression. Among the many genomic responses to merging and/or doubling, changes in the activity of transposable elements (TEs) seem to play a key role in the adaptation to different stresses by modification of the expression of stress-related genes (Quadrana et al., 2019). Environmental changes or stress can result in the proliferation of highly mutagenic TEs due to a transient relief of gene silencing (Kashkush et al., 2003; Madlung et al., 2005; Tittel-Elmer et al., 2010; Lopes et al., 2013; Willing et al., 2015; Springer et al., 2016; Edger et al., 2017), and also to the presence of specific activator sequences in TE promoters (Galindo-González et al., 2017). Particularly, genomic TE content frequently increases after polyploidization events, usually affecting specific TE families which may be more susceptible to activation (McClintock, 1984; Yaakov and Kashkush, 2012). While genome shock caused by interspecific hybridization has been considered as the main trigger of changes in genomic (and conversely TE) dynamics (see Hegarty et al., 2006; Parisod et al., 2009), long terminal repeat (LTR) retrotransposons have been shown to be activated following WGD also in autopolyploids (Bardil et al., 2015). TEs contain internal promoters to facilitate their own expression, therefore, the insertion of TEs within or close to genes can alter the expression of neighboring genes by providing additional transcription factor binding sites or alternative promoters and splicing signals (Lisch, 2013). Interestingly, TE integrations are frequently not random, and it has been shown that some LTR-retrotransposons preferentially target environmentally responsive genes (Quadrana et al., 2019), generating new genetic or epigenetic variability that could facilitate adaptation to stressful conditions. In particular, TE-driven upregulation of stress-responsive genes has been reported in the case of aluminum tolerance genes, as well as in genes involved in the response to diseases in pepper and wheat (reviewed in Blasio et al., 2022), among others (see also below).

An interestingly feedback regulation between polyploidy-induced epigenetic modifications and stress-responsive gene expression changes has been recently proposed by Wang et al. (2021), as a general mechanism for polyploid plants and crops to respond and adapt to environmental and climate challenges during evolution and domestication. In this context, it is worth mentioning that stress response in plants involves priming, which is an adaptive strategy to increase survival capacity in response to rapidly changing environments. Upon stress, plants can evoke many changes at physiological, metabolic, transcriptional, and epigenetic levels, which allow them to augment responses that help increase stress tolerance (e.g., Schwachtje et al., 2019). Particularly, changes in DNA methylation have been involved in priming by altering the expression of TEs proximal to stress-related genes, whose transcription in turn was affected, altering plant susceptibility against biotic stress (e.g., Le et al., 2014). In line with these findings, Wang et al. demonstrated that, in rice plants, polyploidy may repress proximal TEs through histone modifications, leading to CHH hypomethylation by the RNA-directed DNA methylation (RdDM) pathway. Under salt stress (the first salt treatment), CHH hypomethylation “primes” stress-related genes, including those in jasmonic acid (JA) biosynthesis and related signaling pathways, for more rapid and stronger activation in tetraploid than in diploid plants, thus improving salt-stress tolerance in the former. In turn, highly expressed stress-related genes may derepress neighboring TEs, triggering CHH hypermethylation to further silence these TEs. After stress removal, the abundance of JA-related gene transcripts in tetraploids returns to a level similar to that in diploid plants, while the hypomethylated state of CHH from tetraploids before the first salt treatment is also partially retained. After recurrent salt stress, tetraploid stress-related genes are more strongly induced and subsequently hypermethylated in a process similar to the one mentioned above, thus enhancing their adaptation to the imposed stress condition.

In addition to providing new regulatory sequences, TEs can also be the source of small RNAs (sRNAs) that affect gene expression (Nieto Feliner et al., 2020). Small RNAs, including microRNAs (miRNAs) and different classes of small interfering RNAs (siRNAs) are endogenous 21–22 nt-long RNAs which play an essential role in regulating gene expression in different developmental processes, by targeting mRNAs for repression at the transcriptional and post-transcriptional level (Bartel, 2004; He and Hannon, 2004; Khraiwesh et al., 2012). MicroRNAs involved in the regulation of the expression of stress-related genes in a protective fashion have been detected in different plant species. Notably, when plants undergo WGD, miRNA families expand (Maher et al., 2006; Ha et al., 2009; Kenan-Eichler et al., 2011), and the study of the differential expression of known miRNAs or the detection of novel miRNAs in polyploids could be crucial for the understanding of how polyploidy can render species more tolerant to stressful environments.

Considering the significant challenges humanity is facing in relation to food supply and climate change, understanding the role that polyploidy plays in enhancing plant tolerance to various types of stress and in expanding the range of conditions for plant establishment may lead to better breeding and crop-improvement strategies. In the next sections we will review the current literature regarding the association between plant polyploidy and tolerance to different abiotic and biotic stresses, with an emphasis on the distinct mechanisms responsible for these effects, where available. We will examine whether WGD may be directly related to improved stress tolerance and we will evaluate the impact of ploidy level on plant survival and production, particularly in crop species with intrinsic agronomic value or that are essential for different industrial processes. Given the wide range of environmental stress factors faced by plants, we focused on those factors in which the most progress has been made in the knowledge of the putative morpho-physiological and/or molecular mechanisms involved, revealing aspects in common, as well as remaining questions that need to be addressed in future research. A summary of the cited as well as other related studies and their primary conclusions can be found Supplementary Tables 1–5.

## Genome duplication and abiotic stress tolerance

### Water deficit stress

#### Impact of water deficit stress

Water availability is one of the main environmental factors limiting growth and development in plants (Bohnert et al., 1995; Bray, 1997; Gupta et al., 2020), rendering drought tolerance a major challenge for agriculture and ecosystem health, especially in the context of climate change and increasing food demand, and making this kind of abiotic stress the most studied worldwide. The fact that a doubling of the drought risk for world crop production has been predicted (Li et al., 2009b), emphasizes the need to design new crop management techniques and the development of more drought tolerant plant varieties. Drought is a difficult term to define but, in general, plants will be under water deficit stress when the available soil water is insufficient to meet their demand. This imbalance can be dynamic and may provoke short-term physiological acclimation responses or lead to long-term adaptive mechanisms (Chaves et al., 2003; Verslues et al., 2006). The latter may involve drought avoidance strategies, such as early flowering, in order to complete the life cycle before the start of the dry season. In the short term, water deficit affects primarily the physiological processes of transpiration and photosynthesis, and, because it influences cell expansion, cell division, plant hydraulics, hormone homeostasis and gene expression, it can lead to long-term changes on vegetative growth and reproductive development (Tardieu et al., 2018). The magnitude and consequences of the damage due to water deficit will depend on the time of initiation, duration and intensity of the drought. In crops, when it occurs during the reproductive stage, drought can result in yield losses of more than 40% (Boyer, 1982).

The evidence strongly indicates that polyploidy promotes drought tolerance in plants (see Supplementary Table 1). However, the literature is not conclusive in terms of a single mechanism by which WGD can lead to such tolerance. Some component of it is most certainly due to the larger cell size caused by polyploidy, affecting not only the size of the roots for water absorption and the thickness of leaves for water release, but also the size and number of stomata and of xylem vessels, which together control the capacity for water movement in the continuum soil–plant-atmosphere, and hence, the plant’s response to water deficit. On the other hand, transcriptomic results point to gene dosage or gene transcription effects on the content of abscisic acid and its signaling components, metabolic changes leading to osmolyte accumulation, as well as an increase in antioxidant capacity, allowing the polyploid plant to cope with the higher risk of oxidative damage caused by water deficit and closed stomata.

In the following sections we examine the available information regarding the effect of polyploidy on the main processes and traits that are known to be involved in the response to water deficit.

#### Anatomical changes and resource allocation

Plants deploy multiple strategies in response to water deficit stress to attain different degrees of tolerance (Bohnert et al., 1995; Gupta et al., 2020). Most tolerant plants respond to drought by reducing the rate of water loss, either physiologically by decreasing stomatal conductance or anatomically by increasing the leaf cuticle thickness or by reducing the exposed leaf area, for example through leaf rolling or abscission or by increasing mesophyll thickness. Probably owing to their larger cell size, polyploid plants are known to constitutively show some of those alterations leading to tissue water conservation, a fact that could partially explain their observed drought tolerance. For example, Li et al. (1996) reported a significant increase in the thickness of the lower and upper leaf epidermis and a higher degree of pubescence in drought-tolerant pentaploid and hexaploid *Betula papyrifera*, compared to the diploid. Similar observations were made when comparing tetraploid *Lonicera japonica* (Li et al., 2009a) and tetraploid apple (Wójcik et al., 2022) with their respective diploids, and in these cases the tetraploids also showed a thicker palisade tissue.

Another strategy by which plants maintain a balance between water uptake and transpiration entails modifying the resource allocation from shoots to roots, by developing a deeper root system which enables them to explore larger soil volumes (Poorter et al., 2012). Reports on the impact of polyploidy on root biomass or architecture are very scarce, and hence make it impossible to generalize. A significant constitutive increase in root/shoot biomass ratio has been shown in tetraploid *Citrus limonia* (Allario et al., 2011), which could lead to enhanced drought tolerance, however, to our knowledge this has not been tested. In *A. thaliana* tetraploids, a 20% root biomass increase has been observed, although in this case the effect was dependent on culture conditions (Del Pozo and Ramirez-Parra, 2014) and the tetraploid’s tolerance to water deficit may have been conferred by hormonal rather an anatomical changes (see below).

#### Water transport capacity

Plants subjected to water deficit also respond by altering hydraulic conductivity, either through the modification of xylem conducting properties and suberization of the endodermis, or by regulating the expression and function of aquaporins, which are membrane intrinsic proteins that control the cell’s water permeability and whose transcription and activation respond to environmental cues (Steudle, 2000; Ehlert et al., 2009; Chaumont and Tyerman, 2014; Tardieu et al., 2018). The impact of polyploidy on cell size can lead to increased xylem vessel diameter, which can improve the hydraulic conductivity of the tissue (Ruiz et al., 2020). But even though larger xylem vessels can be more efficient in water transport, they are also more vulnerable to cavitation under drought. In *Chamerion angustifolium*, a herbaceous perennial with natural diploid and tetraploid genotypes occupying different ecological niches, Maherali et al. (2009) found that the drought tolerant tetraploids had larger xylem vessels and higher xylem hydraulic conductivity than diploids, which would allow the former to deplete soil moisture more efficiently before the decrease in soil water potential induces stomatal closure. In contrast, Guo et al. (2016) have shown that *C. angustifolium* natural hexaploids, which also display larger xylem conduits than the diploid, were less tolerant to drought, presumably due to a higher susceptibility to the risk of embolisms caused by water deficit. Thus, at the moment nothing conclusive can be stated about the effect of increased vessel diameter on the drought tolerance reported for some polyploids.

The bigger cell size and more active metabolism of polyploids (Doyle and Coate, 2019) can lead to roots with thicker cortexes or more heavily suberized endodermis, both factors that limit the radial hydraulic conductivity of roots and diminish water uptake, delaying plant mortality under drought by conserving the resource in the soil for longer periods (Steudle, 2000). Allario et al. (2011) reported a doubling of the root cortex width in tetraploid *Citrus limonia*, but provide no information on whether this correlates with tolerance to water deficit. On the other hand, the presence of thicker suberin in the root endodermis has been correlated with enhanced tolerance to water deficit in tetraploid citrus rootstocks (see Ruiz et al., 2020 and references therein).

Regarding the potential influence of polyploidy on water transport mediated by aquaporins, whole-genome transcriptomic studies have not revealed significant changes in aquaporin gene expression upon drought stress in *Citrus limonia* (Allario et al., 2011, 2013) nor *A. thaliana* (Del Pozo and Ramirez-Parra, 2014), nor in apple polyploids (Wójcik et al., 2022). Moreover, Zhang et al. (2015) reported that, while the aquaporin genes *MdPIP1: 1* and *MdTIP1: 1* were upregulated in leaves of apple seedlings subjected to water deficit, their expression levels were consistently lower in tetraploid than in diploid genotypes. Thus, the evidence to date does not suggest that aquaporins mediate the cases of drought tolerance conferred by polyploidy.

#### Regulation of osmolyte content

At the cellular level, plants can maintain turgor under water deficit conditions by accumulating osmotically active metabolites in a process known as osmotic adjustment. Most of the literature on polyploids and water deficit do not provide measurements of osmolyte accumulation but rather report the leaf relative water content, which is an indirect indicator of osmotic adjustment in tissues (Mullan and Pietragalla, 2012). Several authors reported on higher relative water content in polyploids in comparison with diploids, either when well-watered (Tal and Gardi, 1976) or under water deficit conditions (Xiong et al., 2006; Allario et al., 2011; Del Pozo and Ramirez-Parra, 2014; Zhang et al., 2015). In agreement with this notion, Khalid et al. (2021) and Lourkisti et al. (2022), observed an enhanced accumulation of osmolytes under water deficit in different polyploid citrus species. Another, older study on naturally occurring *Betula papyrifera* polyploids showed that they were more capable of maintaining turgor at low tissue water potentials than the corresponding diploids, although the authors did not provide direct evidence on the existence of osmotic adjustment (Li et al., 1996).

#### Stomatal function and transpiration rates

Stomatal pores are the main sites of water loss at the whole plant level. While stomatal closure helps to conserve water, it can also impose limits to photosynthetic CO_2_ uptake, thus leading to decreased biomass accumulation and crop yield (Tardieu et al., 2018). Changes in stomatal conductance as well as in anatomical features like stomatal size, density and/or distribution have been proposed as the most important mechanisms of control of water use efficiency (defined as the amount of carbon gained relative to the amount of water used; see Hetherington and Woodward, 2003; Lawson and Blatt, 2014; Franks et al., 2015; Leakey et al., 2019). Stomatal movements are driven by changes in turgor pressure, a process that is under the control of the hormone abscisic acid (ABA), among other factors (Wilkinson and Davies, 2002).

Stomatal size and density vary within species and are controlled in part by environmental cues, including light quality/intensity, and CO_2_ partial pressure (Faralli et al., 2019). Lower stomatal densities correlate with lower transpiration rates, and significant improvement of water use efficiencies have been demonstrated in transgenic plants with fewer stomata (Franks et al., 2015; Hughes et al., 2017). Regarding polyploidy, it is widely accepted that genome size correlates positively with stomatal size and negatively with stomatal density (Beaulieu et al., 2008), and fewer and larger stomata have been found in most, if not all, polyploids analyzed to date. The observation that polyploidy and reduced transpiration rates may be linked was made early on (Chen and Tang, 1945; Tal and Gardi, 1976), and many reports show that this in turn could have a positive impact on tolerance to water deficit by delaying wilting (Supplementary Table 1; Allario et al., 2011, 2013; Del Pozo and Ramirez-Parra, 2014). Most of the available evidence to explain how the lower transpiration rates come about in polyploids points to two main factors: fewer and larger stomata on the leaf epidermis and differential levels of ABA accumulation, transport or sensitivity. It has also been shown that the size of stomata influences the kinetics of stomatal movements, probably by affecting surface-to-volume ratios of guard cells and the efficiency of solute transport to drive movement, with smaller stomata responding more rapidly to environmental or hormonal stimuli (Drake et al., 2013). However, comparative information on stomatal kinetics for the larger stomata of polyploids is very limited, with one report in *A. thaliana* showing that natural and synthetic tetraploids, although possessing stomata of similar size, differed in stomatal kinetics and overall conductance (Monda et al., 2016).

#### ABA homeostasis

The regulation of transpiration by ABA involves long-distance transport from roots to shoots, and local modulation of ABA concentration at the stomatal guard cells (Wilkinson and Davies, 2002; Chaves et al., 2003; Kuromori et al., 2018). Besides the structural effect that lower stomatal densities can have on stomatal conductance and water conservation, several lines of evidence also highlight the existence of an altered ABA homeostasis in polyploids, suggesting that changes in ABA regulation may be related to their enhanced tolerance to drought. In *A. thaliana*, genome duplication does not appear to cause significant differences in ABA concentration *per se* (Monda et al., 2016), however, several reports have shown a constitutive upregulation in tetraploids of a number of genes involved in ABA metabolism and signaling, as well as other genes involved in the response to drought (Del Pozo and Ramirez-Parra, 2014, 2015; Li et al., 2017). Moreover, although several drought-induced genes (bearing ABA-responsive motifs) are greatly upregulated under drought treatment in both diploid and tetraploid genotypes, some of these transcripts are over-represented in the absence of drought in tetraploids (Del Pozo and Ramirez-Parra, 2014), suggesting that, at least in this species, polyploids may be primed for stress tolerance by having a pre-activated ABA response. Similarly, and compared with the corresponding diploids, constitutively increased ABA concentrations were found in roots of tetraploid *Citrus limonia* (Allario et al., 2013) and in leaves of tetraploid *Lycium ruthenicum* (Rao et al., 2020). Since whole-genome transcriptomic comparisons of diploids with their polyploids grown under normal growing conditions have been restricted to these few species, it is not possible at the moment to generalize about such a priming effect.

#### Antioxidant capacity

Severe water deficit conditions can affect cellular redox homeostasis and lead to symptoms of progressive oxidative stress (Tardieu et al., 2018). Low internal CO_2_ partial pressure in leaves of droughted plants (due to closed stomata) can increase the accumulation of reactive oxygen species (ROS) derived from overreduction of the photosynthetic electron transport chain, and from the stimulation of the photorespiratory pathway. A frequently reported response of plants exposed to severe drought (and other types of stress, see below) involves changes in the expression and activities of major antioxidant enzymes such as superoxide dismutase (SOD), catalase (CAT), and/or ascorbate and guaiacol peroxidases (APX and POD), as well as changes in key ROS scavengers such as ascorbate and glutathione (Pan et al., 2006; Noctor et al., 2014; Demidchik, 2015). Several reports point to a higher antioxidant activity in polyploids. Yang et al. (2014) showed that the improved drought tolerance of two lines of colchicine-induced rice tetraploids correlated with increased antioxidant capacity when compared to the diploid line. Similar results were reported by Deng et al. (2012) when comparing *Nicotiana benthamiana* octaploids and diploids subjected to similar degrees of water deficit. An increased ROS detoxification capacity was also found in tetraploid *A. thaliana* (Li et al., 2017). More recently, this enhanced antioxidant capacity was reported in synthetic autotetraploid apple, which showed higher APX expression than the diploid under drought (Wójcik et al., 2022), and in autotetraploid *Ziziphus jujuba*, where the higher activity of antioxidant enzymes correlated with a lower degree of oxidative damage to cell membranes (Li et al., 2021). Taken together, these findings suggest the involvement of gene dosage or other gene-expression altering effects in the regulation of antioxidant capacity. However, the apparent relationship between ROS detoxification efficiency and the expression of genes involved in the antioxidant response upon drought exposure in polyploids is far from being resolved. Del Pozo and Ramirez-Parra (2014) performed a whole transcriptome expression analysis in *Arabidopsis* and showed that tetraploidy enhances the expression of genes involved in the response to oxidative stress under well-watered conditions, irrespective of the ecotype considered. However, when plants were subjected to drought, the same genes, and also ABA responsive genes, were down-regulated in the tetraploids compared to diploids (Del Pozo and Ramirez-Parra, 2014). Similarly, in a series of experiments performed on *Citrus limonia* diploid and autotetraploid lines, the enhanced drought tolerance of the tetraploid was not associated with large changes in gene expression levels between genotypes, with genes participating in ROS detoxification being down-rather than up-regulated in the tetraploid when compared to the diploid line (Allario et al., 2011, 2013). These results may seem contradictory, but the underlying cause of drought stress tolerance in polyploids may be that gene duplication and gene dosage lead to constitutively higher content of ABA, ROS scavengers and ROS detoxifying enzymes, which may in turn raise the threshold at which the polyploid genotypes start to experience drought stress and express drought-related genes. It is also important to keep in mind that, generally, the comparisons were made in experiments designed not to provide the same degree of water deficit (i.e., same soil water potential) to the contrasting genotypes, but rather after a fixed time period following the cessation of normal watering. Thus, data interpretation may be confounded by the fact that polyploids with low transpiration rates consume less pot water than their diploid counterparts, and thus delay the onset of the soil water deficit necessary for the display of stress symptoms. Controlled drought experiments in which gene expression is evaluated simultaneously with changes in soil water potential are necessary to solve this conundrum.

### Temperature stress

Temperature is a key determinant for the geographic distribution of organisms. Particularly, plants can be found within an approximate thermal range of −10°C to 60°C, defined by the freezing point of intracellular water and the temperature of protein denaturation (Fitter and Hay, 2002). Depending on the species, there is an optimum temperature range where growth and developmental processes occur at the highest rate, and beyond which plants should display different responses to cope with either the cold or the heat stress imposed.

Temperature-associated stress affects several developmental processes along the life cycle of plants (Hedhly, 2011). Chilling temperatures, for instance, thermodynamically lower the fluidity of biomembranes and cause marked disturbances in many physiological processes such as water and nutrient uptake, photosynthesis and respiration, all of which severely impair plant productivity (Jouyban et al., 2013). On the other hand, high temperatures can also alter membrane permeability, protein and cytoskeleton stability and chromatin structure, negatively affect respiration and photosynthesis, and impact on floral development and pollen viability, thus decreasing overall plant growth and/or fruit or seed yield (Bita and Gerats, 2013; Dwivedi et al., 2017; Rieu et al., 2017). In the case of crops, it has been reported that the impact of one degree-Celsius increase in global mean temperature would reduce maize, rice, wheat and soybean yields between 3.1% and 7.4% (Zhao et al., 2017a). Given the present context of global warming, strategies aimed to improve plant tolerance and crop yield in the face of increasing temperatures are needed, which makes polyploidy a promising alternative (see Supplementary Table 2).

In the following sections we summarize the research works addressing the effect of polyploidy on plant responses to either cold or high temperature stress, and then we discuss the advances in the knowledge of the possible genetic and/or molecular underlying mechanisms.

#### Cold stress responses

Different lines of evidence suggest that polyploidization is associated with the expansion of gene families involved in the cold temperature response, an association usually related to the direct effects of cold on diploid and polyploid gamete formation (te Beest et al., 2012). Since cold temperatures may contribute to the formation of polyploidy, cold tolerance may be important for the survival of newly formed polyploids. Increases in cold stress tolerance have been reported for polyploids of several plant species such as *Hedyotis caerulea*, *Lolium perenne*, *Nicotiana benthamiana, Plumbago articulata,* among others (Sugiyama, 1998; Huang et al., 2007; Deng et al., 2012; Jiang et al., 2020). In colchicine-induced octaploids of *N. benthamiana*, survival time under cold stress increased about 70% when compared to tetraploids, apparently due to an enhancement in oxidative stress management by means of lower H_2_O_2_ production and a higher ROS scavenging capacity, both under stressful and stress-free conditions. In addition, octaploids showed a higher net rate of photosynthesis than tetraploids under decreased endogenous CO_2_ availability (Deng et al., 2012).

A synthetic tetraploid of the ornamental/medicinal shrub *Plumbago auriculata* also showed higher tolerance to a short-term cold stress treatment (24 h, 5°C) than its diploid counterpart (Jiang et al., 2020). This response was associated to anatomical changes in root and, particularly, leaf tissues in the tetraploid (e.g., thickened spongy mesophyll, widened leaf stomata, and an increased guard cell size), which contributed to enhance photosynthetic activity (as estimated by chlorophyll fluorescence parameters) under cold stress. On the other hand, the tetraploid genotype showed a significantly lower level of lipid peroxidation than the diploid, suggesting a better stability of the membrane system, probably due to an enhanced ROS-scavenging capacity.

The correlation between ploidy level and enhanced temperature-stress tolerance is not always straightforward. Lagibo et al. (2005) conducted a comparative analysis of frost tolerance in garden pansy (*Viola × wittrockiana* Gams) among different genotypes with multiple levels of ploidy (10x, 14x, and 16x), under field conditions. Two standard octaploid cultivars were used as controls. The authors analyzed changes in pigment content and used chlorophyll fluorescence parameters to evaluate plant tolerance to low and sub-zero temperatures. While a positive correlation was found between ploidy level and pigment production (which is suggestive of greater photochemical activity in polyploids), the genotypes under study responded to frost independently of their ploidy level: hexadecaploids were ranked as sensitive to intermediate, followed by 12x (sensitive), while 10x and 14x genotypes were as tolerant as the 8x controls. These results strongly suggest that physiological and/or molecular mechanisms other than pigment production should be evaluated to better assess the effect of ploidy level on frost tolerance in this species.

#### Heat stress responses

Regarding heat stress, an increased tolerance to supraoptimal temperatures has been also reported in polyploids of different plant species. For instance, *Dioscorea zingiberensis* tetraploids show enhanced tolerance to high temperatures, in part due to a higher content of antioxidants (particularly ascorbic acid and glutathione) and higher antioxidant enzyme activity than in the diploid (Zhang et al., 2010). Xu et al. (2011) further showed that this species responds with a ploidy-dependent pattern of transcriptomic changes under high temperature conditions, which might contribute to the evolutionary success of polyploids in warmer niches (see Section “Advances in the knowledge of genomic and molecular mechanisms underlying temperature-stress tolerance in polyploids”).

Similarly, in a study aimed at comparing the effect of ploidy level on morphological traits, metabolite content, and heat stress-associated gene expression in the medicinal crop *Cnidium officinale*, Kim et al. (2021) reported that synthetic autotetraploids had a higher tolerance to a heat-shock treatment compared to diploids (measured as a lower increase in the expression of a heat-stress marker). Although the mechanistic basis for this response was not elucidated, the higher thermal resistance of the autotetraploid could be related to an increased content of phenolic compounds with strong antioxidant activity (particularly naringin, salicylic acid and gentisic acid) as a result of polyploidization.

Finally, Wei et al. (2020) failed to observe the increase in heat tolerance usually associated with polyploidy, when comparing synthetic autotetraploids and their respective diploids from two wild *Fragaria vesca* subspecies. The authors suggest that their results can be explained by a resource allocation toward growth and clonal reproduction in the greenhouse environment where the trial was conducted. In those conditions, the observed elevated cell injury under heat-stress in the polyploids was positively correlated with the increase in clonal reproduction in response to genome doubling. This clearly reinforces the idea that stress tolerance following genome doubling is not only taxon-but also environment-dependent.

#### Advances in the knowledge of genomic and molecular mechanisms underlying temperature-stress tolerance in polyploids

Stressful conditions can have considerable effects on the expression of duplicated genes after a WGD event. This might lead to an overall transcriptional reprogramming enabling a phenotypic plasticity that confers an adaptive advantage to polyploids. For instance, Dong and Adams (2011) demonstrated that heat treatment induced differential expression of three specific homologous genes. In a similar line of evidence, Liu and Adams (2007) reported that only one of the alcohol dehydrogenase homologs in allopolyploid cotton was specifically expressed under cold stress. More recently, Lee and Adams (2020) conducted a thorough transcriptomic analysis on *Brassica napus* (a natural allotetraploid) under cold, heat and drought conditions, and found that stress (especially cold stress) favored gene expression in one parental subgenome (*B. rapa*), while alternative splicing alterations were more frequently found on the other parental subgenome (*B. oleracea*). These results are suggestive of a process where stressful thermal conditions could be a driving force for the preservation of certain homologs in polyploids *via* subfunctionalization.

In an attempt to deepen the knowledge of the molecular mechanisms underlying the increased tolerance to high temperatures in *D. zyngiberensis* polyploids (see above), Xu et al. (2011) compared transcriptomic changes through sequence-related amplified polymorphism (SRAP)-cDNA and SRAP analysis in diploid and synthetic autotetraploids, prior and after heat-stress. The study revealed that tetraploids developed an “activation transcriptome response” pattern (i.e., a higher proportion of overexpressed transcripts over silenced ones) under high temperature stress, in contrast to a “random transcriptome response” pattern (i.e., a similar ratio of silenced to activated transcripts) found in diploids. According to SRAP analysis, the differences in expression patterns were not due to genetic changes after WGD, but rather to epigenetic and/or post-transcriptional regulatory mechanisms. Although the functional role of the affected genes was not elucidated, it is suggested that the observed ploidy-dependent pattern of transcriptomic response might in part explain the higher temperature tolerance reported for tetraploids. Further research will be necessary to test this hypothesis as well as to reveal more details regarding the molecular mechanisms involved.

In a study on the sequenced genomes of representative species of the Brassicaceae and several model plants (including monocots such as *Oryza sativa* and *Zea mays*, the basal angiosperm *Amborella trichopoda,* the lycophyte *Selaginella moellendorffii* and the moss *Physcomitrella patens*), Song et al. (2020) performed comparative analyses of cold-regulated genes (*CRGs*) to explore their retention, positive selection, expression and involvement in regulatory networks after polyploidy. The authors found that in 17 of the 21 plant species analyzed, polyploidization was the predominant mechanism by which *CRG* homologs originated, accounting for 46%–96% of these homologs in each genome. Most *CRGs* belong to transcription factor families, and their regulatory networks were much larger in plant genomes affected by more polyploidization events. Based on their analyses, the authors conclude that polyploidy plays an important role in resisting the stress imposed by cold. Nevertheless, and despite the available evidence (summarized in Supplementary Table 2), investigations on the underlying molecular mechanisms for increased cold/heat tolerance in polyploids is still scarce, and further research is essential for a better understanding of the role of WGD in this phenomenon.

### Salinity stress

#### Impact of salt stress

Soil salinization is a serious global threat to agricultural production (Ivushkin et al., 2019), especially in arid and semi-arid areas (Shannon, 1998; Martinez-Beltran and Manzur, 2005). At present, salinity affects more than 1 billion hectares of cultivated land, distributed all over the world and involving more than 100 countries (FAO and ITPS, 2015). According to the Fifth Assessment Report of the United Nations Intergovernmental Panel on Climate Change (AR5-IPCC 2015), larger areas will be affected by salinity-related problems in the near future, due, among other causes, to the rise in sea level. In this context, the development of crops more resistant to salinity stress will be fundamental to sustain agricultural production. Different publications show that natural or induced polyploids of some species are more tolerant to salinity stress than diploids (Supplementary Table 3). Thus, polyploidization may have an important role in the development of crops more resistant to this type of stress.

In the following sections we discuss the main physiological mechanisms involved in the acclimation or adaptation responses to salinity stress, and we then discuss recent advances in the knowledge of the genetic and/or molecular processes underlying the effect of polyploidy on salinity stress tolerance.

#### Salt stress response mechanisms

Among the multiple negative effects caused by salinity stress, the most relevant are water deficit, membrane permeability alterations, ion toxicity, nutrient deficiency and free radical imbalances, all of which affect plant growth, morphology, and survival (Zhu, 2001; Golldack et al., 2014; Zhang et al., 2014; Lu et al., 2017). It is worth noting that several adaptations found in halophytes can also be found in both natural and induced polyploid plants (Supplementary Table 3). Plants use different physiological mechanisms to neutralize Na^+^ toxicity, such as limiting its entry into the roots, shuttling it from the cytosol to the apoplastic space, actively accumulating Na^+^ into vacuoles, or controlling Na^+^ transport in the xylem (Kumari et al., 2015; Mishra and Tanna, 2017). In general, plants that can maintain an adequate cellular ionic balance, but particularly a high K^+^/Na^+^ ratio, are more tolerant to salinity (Munns and Tester, 2008; Pan et al., 2016). This is particularly evident in *A. thaliana,* where a study involving numerous accessions, including haploid genotypes, natural diploids and natural or induced autotetraploids, showed that ploidy was a significant determinant of leaf K^+^ concentration (Chao et al., 2013). Moreover, under moderate salt stress conditions (200 mM NaCl), the increment in leaf K^+^ concentration in tetraploid leaves was accompanied by a decrease in Na^+^ accumulation, thus enhancing both K^+^/Na^+^ ratio and salinity tolerance in these genotypes (Chao et al., 2013). This salinity tolerance is not limited to autopolyploids. Polyploid species of the genus *Brassica* were also shown to be more salt-tolerant than their diploid ancestors; this was shown both in allotetraploids (Meng et al., 2011) and autotetraploids (Ashraf et al., 2001).

#### The role of root cells

Using a reciprocal grafting technique, Rus et al. (2006) showed that *A. thaliana* root cells control leaf K^+^ concentration and, consequently, the tolerance to saline stress. Similarly, Chao et al. (2013) demonstrated that the ploidy level of root grafts can exert control over the leaf K^+^ concentration regardless of the ploidy level of the rest of the plant. Even though the mechanism involved has not been clearly elucidated, polyploid rootstocks can be used to increase tolerance to saline stress while maintaining the desired agronomic traits of diploid grafts. An example of this can be found in citrus crops, where the capacity of polyploid rootstocks to exclude Cl-from leaves increased the tolerance to moderate salinity stress (Bañuls et al., 1990; Bañuls and Primo-Millo, 1992, 1995; Saleh et al., 2008).

Reducing the outflow of K^+^ induced by NaCl in root cells may contribute to maintain K^+^/Na^+^ homeostasis in this organ (Shabala and Pottosin, 2014). Potassium outflow from root cells is mediated by three different types of channels: depolarization-activated outward-rectifying K^+^-permeable channels (DA-KORCs), weakly voltage-dependent non-selective cation channels (NSCCs) and reactive oxygen species (ROS)-activated K^+^-permeable channels (including KORCs and NSCCs; Shabala et al., 2016). Another possible mechanism to maintain K^+^/Na^+^ ratio under salinity stress is Na^+^ exclusion from the cytosol, which is mediated by a plasma membrane Na^+^/H^+^ antiporter, named salt overly sensitive 1 (SOS1), whose activation is mediated by Ca^2+^ (Sun et al., 2009; Maathuis, 2014; Zhu, 2016). ROS also have an important role in the regulation of Na^+^ homeostasis through the stabilization of SOS1 mRNA (Chung et al., 2008), as well as the increase of cytosolic Ca^2+^ concentration due to the activation of plasma membrane Ca^2+^-permeable channels (Sun et al., 2010; Ma et al., 2012) and the increase in the activity of plasma membrane H^+^/ATPase (Zhang et al., 2007; Niu et al., 2018). Polyploidization was shown to affect many of these processes, and, as mentioned above, strategies aimed at maintaining the cellular ion balance, a high K^+^/Na^+^ ratio, and low levels of toxic ions in leaves and meristematic tissues appear to be a hallmark of such adaptations. Depending on the species, this may involve the regulation of the expression, the activity and/or the sensitivity of specific ion channels in order to particularly facilitate either Na^+^ exclusion, K^+^ and Ca^2+^ absorption or retention, as well as to regulate Ca^2+^- dependent signaling pathways (Lu et al., 2006). A summary of the findings reported so far on the impact of ploidy levels on some of these mechanisms (as well as others, see below) can be found in Supplementary Table 3 references therein.

#### Antioxidants and osmoregulatory metabolites

Another adaptation that plants present to prevent the effects of salinity stress is to stimulate the production of osmotically active metabolites and of enzymatic and/or non-enzymatic antioxidants aimed to counteract the increment of ROS (Hasegawa et al., 2000; Yamada et al., 2005; Munns and Tester, 2008). Under salt stress, several polyploid species showed significant differences in the production of key antioxidants such as glutathione and proline, as well as greater superoxide dismutase (SOD) and peroxidase (POD) activity than the diploid controls (Supplementary Table 3). These changes were correlated with lower contents of malondialdehyde (MDA), a commonly used indicator of lipid peroxidation and membrane damage (Liu et al., 2011; Jiang et al., 2013; Tu et al., 2014; Fan et al., 2016a). On the other hand, transcriptomic and metabolomic studies also highlight the upregulation of metabolic pathways involved not only in antioxidant defense, but also in the synthesis of free amino acids (usually proline, but also methionine, aspartic acid, and arginine, among others), organic acids (e.g., oxaloacetic acid, fumaric acid), polyhydric alcohols (myoinositol, inositol), soluble sugars, polyamines, flavonoids and/or late embryogenesis abundant (LEA) proteins (which are rich in hydrophilic amino acids), as biochemical mechanisms to protect cells from dehydration damage, both in salt-tolerant diploids (e.g., Li et al., 2021; Qin et al., 2022 and references therein) as well as in polyploids (Wang et al., 2018; Sicilia et al., 2019; Zhao et al., 2022; see also below).

#### Advances in the knowledge of genomic and molecular mechanisms underlying salinity-stress tolerance in polyploids

Recent comparative proteomic and genomic analysis between polyploid plants and their diploid ancestors under high salinity conditions reveal that proteins involved in numerous processes like photosynthesis, stress and defense, energy, metabolism, transcription/translation, and transport may be differentially expressed among genotypes (Podda et al., 2013; Wang et al., 2013b; Deng et al., 2017). A sugar transporter, a chloroplastic ATP synthase delta subunit, and enzymes involved in photosynthesis and ROS scavenging are among the most relevant found to be upregulated in polyploid genotypes of the species studied. Transcriptomic analyses also support the proteomic results, though differences exist depending on the species and experimental conditions tested. For example, in *Paulownia australis* plants treated with 0.2%–0.6% (i.e., 35–100 mM) NaCl solutions for 15 days, the expression of unigenes related to ion transporters, ROS-scavenging system, proline and soluble carbohydrate synthesis, and transcription factors significantly differed between autotetraploid and diploid genotypes (Dong et al., 2017). On the other hand, in two different transcriptomic analyses performed on leaves of *P. tomentosa* diploid and autotetraploid plants grown at different combinations of salt levels and treatment periods, the differentially expressed unigenes (DEU) were assigned to metabolic pathways such as “plant hormone signal transduction,” “RNA transporter,” “protein processing in endoplasmic reticulum,” “photosynthesis process” and “plant-pathogen interaction,” among others (Fan et al., 2016a; Zhao et al., 2017b). Interestingly, changes in the expression levels of transcription factors related to stress responses, such as NAC, MYB, bHLH, GRAS, WRKY, and AP2/EREBP were particularly substantial (Zhao et al., 2017b). In addition to the above-mentioned processes, changes in the expression of DEUs involved in osmoregulation (mostly coding for LEA proteins), have been reported between polyploid and diploid genotypes of *P. fortunae* (Wang et al., 2018) and various species from the genus *Agave* (Tamayo-Ordóñez et al., 2016) after exposure to salt stress. The accumulation of LEA proteins may improve salinity tolerance, as they have important roles in preventing desiccation damage and might contribute to the upregulation of certain antioxidant enzymes (Dalal et al., 2009; Huang et al., 2018). Finally, Xue et al. (2015) studied the expression of two aquaporin genes (*MdPIP1: 1* and *MdTIP1: 1*) in leaves from diploid and autotetraploid apple trees and found that their expression was significantly higher in the polyploids, which also displayed more tolerance to salinity stress than the diploids (note that this is in contrast with the results observed by Zhang et al. (2015) under drought stress mentioned previously) (see sub-section Water transport capacity in section Water deficit stress). The contribution of aquaporins to salinity tolerance has been demonstrated in transgenic plants with constitutive expression of different aquaporin family members (Sreedharan et al., 2013; Xu et al., 2014).

Changes in the expression of miRNAs appear also to contribute to the enhanced tolerance to salt stress of polyploid genotypes. Fan et al. (2016b) and Liu and Sun (2017) studied the differential expression of miRNAs in tetraploid and diploid leaves of *Paulownia fortunei* and *Hordeum bulbosum*, respectively, under salinity conditions. In *P. fortunei*, 10 conserved and 10 novel miRNAs were found to be differentially expressed between the studied genotypes, and the predicted target transcripts of at least eight of these miRNAs corresponded to genes associated with the response to salinity stress. In a survey of changes in the miRNAome of diploid and tetraploid barley (*Hordeum bulbosum*) plants under control (CK) and salt stress conditions, Liu and Sun (2017) reported that 13 miRNAs were differentially expressed due to the genome duplication *per se*. Of these, nine miRNAs were overexpressed in tetraploid CK compared with those in diploid CK plants, while 4 miRNAs were downregulated in the tetraploid as compared with those in the diploid. Interestingly, five of the miRNAs affected by genome duplications were also associated with salt stress. In fact, among these five miRNAs, four (miR171i, miR479, miR5048-5p and miR6196) were upregulated in tetraploid CK compared with those in diploid CK, but miR171i, miR479 and miR5048-5p were downregulated and miR6196 was upregulated in diploid Na-stressed plants with respect to its control. Interestingly, miR528b-3p was only detected in the tetraploid genotype and was downregulated under the stress treatment as compared with the tetraploid CK. The targets of these miRNAs are involved in protection of the photosynthetic machinery against oxidative stress, osmotic stress-activated phospholipid signaling and salt stress response (Liu and Sun, 2017). In view of the results, the authors concluded that, under salt stress, tetraploids have a more elaborate miRNA–target interaction compared with that in diploids, which can help tetraploids to better deal with salt stress while maintaining normal growth. This would be in agreement with physiological data indicating that *H. bulbosum* tetraploids have a stronger ability to retain water and prevent water loss, resulting in better survival under salt stress.

### Nutritional stress

Like any other living organism, plants rely on an adequate supply of certain inorganic minerals for optimal development and growth. Nitrogen and phosphorus are macronutrients essential for photosynthesis, metabolism, growth and development, seed yield, and protein and nucleic acid synthesis, among other fundamental processes (Day and Ludeke, 1993; Zhang et al., 2018). Potassium is essential for osmotic and ionic equilibrium and stomatal movements, is involved in chlorophyll biosynthesis and photosynthesis, and it can enhance the resistance of crops to drought, disease and cold (e.g., Wang et al., 2013a; see also sections 1.1 and 1.3). Calcium is also fundamental for cell wall stability, ion homeostasis, the activity of different enzymes, and as a secondary messenger in numerous signaling pathways, among other biological functions (Thor, 2019). As well as for macronutrients, an adequate supply of micronutrients such as iron, copper, manganese, zinc, nickel and molybdenum is necessary for the functioning of key metabolic enzymes and redox systems, while others like boron are important for cell wall synthesis, pollen tube elongation and carbohydrate transport (Assunção et al., 2022). If left unattended, soil deficiencies in any of these and other nutrients can have a severe impact on agricultural productivity and on the nutritious quality of foods. On the other hand, mineral excess, particularly of micronutrients, may lead to the development of toxicity symptoms, which in turn negatively affects plant growth and yield (Assunção et al., 2022). Mineral nutrient acquisition is directly dependent on the demand of the plant (which is conditioned by the growth rate and the internal concentration of the nutrient) and water availability. Increased carbon allocation to root growth and upregulation of specific transporters are among the most frequent responses of plants to nutrient shortage (Lambers and Oliveira, 2019).

In the following sections we examine the available information regarding the effect of polyploidy on plant responses to cope with nutrient deficiency or excess, and in Supplementary Table 4 we summarize the main processes and conclusions discussed.

#### Plant responses to mineral deficiency

Although the unambiguous translation of an increased ploidy level into an actual enhancement of the overall tolerance of plants to nutritional stress is not sufficiently supported in every case under study, it is interesting to note that an early work from Grant (1952) reported that the rate of polyploid production (due to poor pairing at meiosis) in *Gilia* hybrids grown in low-nutrient conditions was seven-fold greater than that of plants grown in high-nutrient conditions.

Nitrogen starvation is among the main factors limiting plant growth. On the other hand, the abuse of nitrogen (and other mineral-based) fertilizers was shown to have adverse impacts not only on farming, but on the ecosystem as a whole (Kotschi, 2015; Rahman and Zhang, 2018). Hence, strategies aimed at developing crop genotypes with increased N use efficiency are an important alternative to reduce the amount of soil N supplementation. The link between N uptake efficiency and ploidy level is not an obvious one, since some polyploids show higher N contents than their diploid progenitors, while others do not (Noggle, 1946). Under low-nitrogen levels, N assimilation and shoot accumulation in a synthetic allohexaploid wheat was increased with respect to its tetraploid and diploid parents (Yang et al., 2018). This allohexaploid showed a higher root/shoot biomass ratio, higher H^+^ efflux and higher expression of genes lined to N uptake, which may account for the observed differences in tolerance to N deficiency (see Supplementary Table 4). Leps et al. (1980) reported that, in nodulated alfalfa (*Medicago sativa*), tetraploids had higher N fixation rates than diploids during the first 10 days of growth. Interestingly, after that time, both tetraploid and diploid plants fixed N at equal rates. However, increasing the ploidy of alfalfa plants from tetraploid to octaploid did not result in increased N fixation activity or N content.

The efficiency with which roots acquire other nutrients, such as sulfate and potassium, was found to be higher in wheat and sugar beet (but not in tomato) with higher ploidy level (Cacco et al., 1976), compared with their diploid counterparts. The authors correlated the observed uptake efficiency to the level of environmental adaptation of the plants under analysis. In a screen for *Arabidopsis* mutants showing tolerance to nutrient deficiencies, Kasajima et al. (2010) isolated one line that was tolerant to boron deficiency. Ploidy analysis showed this line to be an autotetraploid, although the original screening population was diploid. Furthermore, independently isolated autotetraploid lines were also tolerant to boron deficiency, suggesting that autotetraploidization improves tolerance to boron shortage.

As for most types of environmental stress, nutrient deficiency can increase the susceptibility to oxidative damage, as nutrients are needed for the maintenance of antioxidant activity and cell redox homeostasis (Kandlbinder et al., 2004). In an attempt to develop new citrus rootstocks that require less fertilizer, Oustric et al. (2019) compared the effect of genotype and ploidy level on tolerance to nutrient deficiency in a group of commonly used citrus rootstocks. The experiment was performed on four citrus diploid (2x) seedlings and their four doubled diploid (4x) counterparts. An allotetraploid form was also included in the study. Four-years old trees of each genotype grown under optimal nutrient conditions were divided into two blocks, and irrigated with either the growing fertilizer solution (control) or irrigation water (nutrient deficiency) for a period of 8 months. After analyzing a series of morpho-physiological traits along the experimental period in order to characterize nutrient deficiency symptoms, antioxidant capacity and the level of oxidative damage in the different genotypes, the authors concluded that autotetraploidization did not systematically improve the tolerance to nutrient deficiency, since only one of the 4x seedlings tested showed a significantly different response when compared to its 2x counterpart. Interestingly, the allotetraploid was found as the only resistant genotype, suggesting that allotetraploidization would be a means for increasing tolerance to nutrient deficiency in citrus rootstocks. A better accumulation and/or remobilization of mineral elements related to photosynthesis activity, together with a better performance of the non-enzymatic and enzymatic antioxidant system would account for the increased resistance/tolerance to nutrient deprivation observed in these polyploids.

#### Plant responses to mineral excess

Tetraploidization can alter root anatomy, which in turn can lead to alterations in the capacity for nutrient uptake. Ruiz et al. (2016) described root abnormalities (shorter length, larger diameter, fewer root hairs, etc.) in tetraploid citrus plants, in comparison to their diploid counterparts (see Supplementary Table 4). While expression of boron transporters was not modified by ploidy, these anatomical root alterations might have been responsible for a decreased boron uptake, leading to boron-excess tolerance in the tetraploid.

In a thorough study by Schlaepfer et al. (2010) native (2x and 4x) and invasive (4x) specimens of *Solidago gigantea* (Asteraceae) were compared in their growth performance and overall response to calcium excess. Diploids grown with additional calcium showed reduced biomass accumulation, whereas tetraploids were not affected. Unfortunately, the physiological and/or molecular mechanism underlying the observed responses remain to be elucidated.

Finally, it is worth mentioning that polyploidization in naturally occurring metal hyperaccumulator plants was shown to expand their habitat (e.g., Prasad and de Oliveira Freitas, 2003; Paape et al., 2020). Hence, the use of either natural or synthetic polyploids could be useful as a genetic tool to develop strategies for the phytoremediation of heavy-metal polluted soils.

### UV-B stress

UV radiation is classified in UV-A, -B and-C, according to its wavelength range. While most UV-C radiation is absorbed by the ozone layer, UV-A and UV-B reach the Earth’s surface and cause cellular damage by triggering photochemical alterations in DNA sequences and the accumulation of ROS (Hideg et al., 2013). UV-B alone is responsible for a 5% annual reduction in global agricultural production (Ballare et al., 2001; Caldwell et al., 2007). Throughout evolution, plants have developed a number of strategies to avoid and/or cope with the damage caused by UV-B radiation. Examples of these are cuticle thickening, the accumulation of pigments that act as “chemical shields” (e.g., flavonoids, betalains), production and/or activation of antioxidant enzymes and metabolites which neutralize ROS, and the upregulation of photolyases which repair cyclobutyl pyrimidine dimers that accumulate in the DNA (Chen et al., 2019). The plant response to UV-B radiation can be triggered either specifically, by activation of the UV-B receptor UVR8, or nonspecifically *via* ROS accumulation (Rizzini et al., 2011; Wu et al., 2016; O'Hara et al., 2019).

There are extremely few publications linking tolerance to UV-B stress and polyploidy. Hase et al. (2006) reported that tetraploid Arabidopsis plants grow significantly more than their diploid counterparts under UV-B irradiation. Given this difference cannot be linked to a differential accumulation of pyrimidine dimers in both plants, DNA repair mechanisms were ruled out as a relevant phenomenon responsible for the relative tolerance observed in tetraploids. *Pachycladon cheesemanii* is an allotetraploid perennial herb closely related to Arabidopsis. When both plant species were cultivated under UV-B radiation, Arabidopsis’ growth was significantly reduced, while the growth of *Pachycladon* was less affected. Chlorophyll content-usually considered a marker of UV-B tolerance-was significantly increased in *Pachycladon* after UV-B treatment while chlorophyll content in Arabidopsis Col-0 was not. Although the expression levels of genes belonging to the UVR8-dependent pathway were mildly increased in *Pachycladon* under UV-B irradiation, those related to the UVR8-independent pathway broadly increased. This suggests the enhanced UV-B tolerance shown by *Pachycladon* could rely on a yet not fully described UVR8-independent mechanism (Dong et al., 2019).

Finally, it is worth mentioning that the synthesis of flavonoids and other UV-B-absorbing metabolites was shown to be augmented in certain polyploids (Talei and Fotokian, 2020; Wu et al., 2020a), and polyploids also tend to have a higher ROS detoxification capacity than diploids under different stress conditions (see for example Sections “Antioxidant capacity”, “Temperature stress”, “Antioxidants and osmoregulatory metabolites,” and “Plant responses to mineral deficiency”).

#### Impact of endopolyploidy in the UV-B response

Endopolyploidy is a general term describing the multiplication of nuclear DNA within the cell. In plants, this takes place *via* several mechanisms, but mainly through the process of endoreduplication, which involves the replication of DNA without intervening cell divisions and it is often closely associated with specific cell types, organs, and developmental stages (Leitch and Dodsworth, 2017). This phenomenon is widespread among plant taxa, and it has been suggested that it may play some role in stress tolerance (Adachi et al., 2011).

UV-B radiation has been identified as a positive climatic predictor for a high incidence of endopolyploidy (Gegas et al., 2014). During the UV-B response, the endocycle regulation is mediated by UVR8 (Wargent et al., 2009) and by the atypical E2F transcription factor DP-E2F-like 1 (E2Fe/DEL1). Radziejwoski et al. (2011) demonstrated that E2Fe/DEL1 is a transcriptional repressor of PHR1, a photolyase involved in DNA repair. Upon UV-B irradiation the expression of E2Fe/DEL1 is downregulated, which in turn favors PHR1 activity. Better DNA repair allows plants to rapidly resume the endocycle, contributing in this manner to UV-B radiation resistance by compensating the stress induced reduction in cell number by ploidy-dependent cell growth.

The *uvi4* (UV-B-insensitive 4) mutation promotes the progression of endoreduplication during leaf and hypocotyl development. Hase et al. (2006) showed that the fresh weight of *uvi4* Arabidopsis mutants grown under supplemental UV-B light is two-fold greater than that of the wild type plants grown in the same conditions. In addition, Gegas et al. (2014), have demonstrated that Arabidopsis accessions with an increased level of endopolyploidy are more UV-B-tolerant (as evaluated by fresh weight gain). Hence, the authors suggest that the endopolyploidization contributes to sustaining plant growth under high incident UV radiation.

Increasing our knowledge on the physiological and/or molecular mechanisms underlying the enhanced UV-B tolerance of polyploids will be of the utmost value both to improve crop production, as well as to extend the range of cultivation of species of interest to either higher altitudes, or to regions where increments in the amounts of UV radiation due to alterations in stratospheric ozone are expected (e.g., Bais et al., 2018).

## Genomic duplication and biotic stress tolerance

Pests, parasites and pathogens like bacteria, fungi, oomycetes, and nematodes, cause biotic stress in plants, leading to disease or low yield. It is estimated that between 20% and 40% of crop yield is lost globally to pests and disease (Douglas, 2018). Among pathogens, biotrophic fungi can colonize different plant organs and cause leaf spots and tumors, whereas necrotrophs can release toxins that kill the host cells. Viral infections generally do not kill the host plant but produce a systemic damage leading to stunted growth and malformations. Parasitic nematodes can cause severe root damage and can also act as vectors for viral transmission (Schumann and D’Arcy, 2006). Insect pests have high agronomic impact on crop yield because they cause direct damage and are also responsible for the transmission of plant diseases. Resistance to pathogens and/or insect herbivores may be affected by polyploidization (see Supplementary Table 5). This trait is interesting not only for the development of new varieties but also from an evolutionary point of view.

Apart from the central role of polyploidization in contributing to the biodiversification of flowering plants, Levin (1983) suggested that newly formed polyploid lineages are more resistant to pathogens than their diploid progenitors. This effect can be quite strong and, in the case of perennial species with recurrent polyploid formation, may last indefinitely, potentially providing a general explanation for the successful establishment of novel polyploid lineages (Oswald and Nuismer, 2007). However, this hypothesis has been challenged by studies on herbivory resistance in sympatrically growing diploid and autotetraploid individuals of *Heuchera grossulariifolia* (Thompson et al., 1997; Nuismer and Thompson, 2001), demonstrating that novel polyploid lineages may not receive a uniform or consistent relief from herbivory in the whole set of insect-plant interactions analyzed (see also below). Therefore the authors conclude that it seems unlikely that the evolutionary diversification and success of polyploids resulted from increased resistance to this type of biotic stress.

Different aspects in the morphology and/or development of polyploids have been related to the differential resistance/susceptibility to biotic stress observed among genotypes. For instance, polyploid *Stenotaphrum* genotypes, typically showing enhanced resistance to the nematode *Belonolaimus longicaudatus*, tend to have thicker primary roots than diploids (Busey et al., 1993, see Supplementary Table 5). Interestingly, tetraploid plants of *H. grossulariifolia* were shown to be more susceptible to the attack of *Greya politella* than the diploids, probably due to a change in their flowering time. In fact, the tetraploids flower earlier and partially overlap the flowering of *Lithophragma parviflorum* (another host of *G. politella*), whereas the flowering of diploids generally does not. Hence, there is more of a chance for some females from the local *L. parviflorum* feeding subpopulations of *G. politella* to lay their eggs in tetraploid *H. grossulariifolia* plants than in the diploids (Thompson et al., 1997).

Metabolic differences produced by autopolyploidy can have profound effects for the development and ecological interactions of plant neopolyploids (Vergara et al., 2016). Alkaloids, terpenes, and other classes of secondary products may confer resistance to pathogens and herbivory (Levin, 1983). Similarly, changes in the concentration of metabolites related to the tricarboxylic acid (TCA) cycle and *γ*-aminobutyric acid (GABA) could have important adaptive consequences for the specific ecology of diploids and their polyploid counterparts (Van de Peer et al., 2017).

Recent advances in the study of the interaction between diploid *Arabidopsis thaliana* and synthetic autotetraploids with the phyllosphere microbiome have shed some light on the mechanisms underlying this interaction. It has been observed that there is no difference in the establishment of the synthetic microbiome, neither its composition nor its diversity is significantly affected with respect to host ploidy. Also, at the gene expression level, autotetraploid plants show active defenses constitutively independent of pathogen colonization, whereas diploid plants show a high number of defense-related genes that are differentially expressed in the presence or absence of pathogens (Mehlferber et al., 2022).

Unfortunately, the information available on the physiological and/or molecular mechanisms underlying most of the reported responses is comparatively scarce or nil, so further experimental approaches will be of great help to draw conclusive inferences regarding the effect of polyploidy on biotic stress tolerance.

## Conclusion

Polyploidy plays an important evolutionary role in natural populations. This role can be attributed to a number of consequences of polyploidization that promote phenotypic and/or fitness alterations, such as mutation buffering, increased allelic diversity and heterozygosity, dosage effects, and sub-or neofunctionalization of duplicated genes. Although some controversy regarding this view still exists (e.g., Arrigo and Barker, 2012; Soltis et al., 2014), the overwhelming abundance of polyploid species in nature strongly suggests that polyploidy indeed confers some degree of adaptive advantage. After polyploidization, far-reaching genetic and epigenetic changes in the genome ensue, such as chromosomal rearrangements, amplification of repetitive sequences, loss of DNA sequences, methylation re-patterning and general chromatin remodeling. There are also additive changes due to heterozygosity and gene dosage. Despite the fact that some of the physiological consequences of these alterations are still far from being fully understood, there is a growing body of evidence advancing plausible mechanistic explanations, which support the notion that polyploidy can help plants to improve their tolerance to a wide range of environmental stressors (see Supplementary Tables). In fact, though generalizations should be made with caution, more than 90% of the reviewed experimental data point toward a net positive impact of ploidy level on the ability of plants to cope with different kinds of stress. This would seem to apply to both auto and allopolyploids, even though differential responses due to the genome duplication process have been reported depending on the type of stress and species considered. In view of this, and considering that certain plant species have been used by different authors as a model to test the role of polyploidy against a variety of stressors, it is tempting to advance the notion that WGD events have the potential to enhance the plant’s ability to cope with environmental stress *in general*. Nevertheless, more systematic studies that perform a comparative analysis of responses to different types of stress in the same species and under similar general conditions (see for example Deng et al., 2012) are necessary, in order to adequately address this notion.

In the face of rapid climatic and other environmental changes at the global scale, understanding the impact of polyploidization in plant evolution and ecological interactions is of an uttermost relevance, as this knowledge might rapidly become an important tool for the breeding of economically important crops, helping us to pave the way for harnessing more efficient uses of artificial polyploidization to obtain genotypes with increased tolerance to diverse biotic and abiotic stresses.

## Author contributions

All the authors made substantial contributions to the conception or design of the work and to the acquisition and interpretation of data for the review. SIP-Á, HFC, and IB agree to be accountable for all aspects of the work in ensuring that questions related to the accuracy or integrity of any part of the work are appropriately investigated and resolved. All authors contributed to the article and approved the submitted version.

## Funding

The work was supported in part by CONICET, Argentina (grant PIP 11220200101935CO, to IB).

## Conflict of interest

The authors declare that the research was conducted in the absence of any commercial or financial relationships that could be construed as a potential conflict of interest.

## Publisher’s note

All claims expressed in this article are solely those of the authors and do not necessarily represent those of their affiliated organizations, or those of the publisher, the editors and the reviewers. Any product that may be evaluated in this article, or claim that may be made by its manufacturer, is not guaranteed or endorsed by the publisher.
