# Supplementary material for: Impact of polyploidy on plant tolerance to abiotic and biotic stresses
Source: Front Plant Sci. 2022 Aug 22;13:869423. doi: 10.3389/fpls.2022.869423 (PMC9441891; doi:10.3389/fpls.2022.869423)
Supplement: Supplementary file 1 [file Data_Sheet_1.docx]

## Supplementary tables

***Supplementary Table 1. Effect of polyploidy on responses to drought stress. Entries were ordered according to the year of publication of the article referenced.***

| **Stress** | **Experimental conditions** | ***Species*** | **Type of polyploidy** | **Polyploidization effect** | **Putative processes involved** | **Reference** |
| --- | --- | --- | --- | --- | --- | --- |
| Drought | Soil drying | *Hordeum vulgare* | Colchicine-induced tetraploids vs. diploid | Increased drought-tolerance | Autotetraploid had slower transpiration rate and higher leaf osmotic potential than diploid | Chen and Tang, 1945 |
| Drought | 2hr simulated drought stress with polyethylene glycol | *Betula papyrifera* | Natural penta- and hexaploid vs. diploid | Increased drought-tolerance | Polyploids have lower stomatal density, thicker leaf epidermis and more dense pubescence than diploids. Pentaploids had better turgor maintenance capacity. Pentaploid retained higher net photosynthesis under water stress. | Li *et al*., 1996 |
| Drought | Potted plants subjected to progressive water deficit | *Triticum aestivum, T. monocccum, T. dicoccum* | Wild (diploid and tetraploid) relatives vs. cultivated (hexaploid) wheat | Increased drought-tolerance | The sensitivity of stomatal conductance to drying soil and the ability to maintain a leaf water content was proportional to ploidy level. Highest sensitivity to a non-hydraulic root signal (ABA?) in 6x modern wheat | Xiong *et al*., 2006 |
| Drought | Withdrawal of watering of potted plants | *Lonicera japonica* | Colchicine-induced tetraploids vs. diploid | Increased drought-tolerance | Tetraploid has fewer, thicker leaves with denser pubescence than diploid, and depletes soil water more slowly. Tetraploid maintains higher stomatal conductance and net photosynthesis longer than diploid after watering withdrawal. | Li *et al*., 2009 |
| Drought | Withdrawal of watering of potted plants | *Chamerion angustifolium* | Natural and synthetic autotetraploids vs. diploid | Increased drought-tolerance | Autotetraploid shows lower stomatal density and increased stomatal length. The number of xylem vessels in the autotetraploid was reduced but their diameter increased, thus enhancing stem hydraulic conductivity. Natural tetraploids showed delayed wilting under drought. | Maherali *et al*., 2009 |
| Drought and other abiotic stresses | Polyethylene glycol treatment of hydroponically grown plants | *Dendranthema nankingense* | Colchicine-induced autotetraploids vs. diploid | Increased stress tolerance and lower oxidative damage | Tetraploid shows increased stomatal size and decreased stomatal density, higher relative water content under stress, and higher peroxidase activity but lower superoxide dismutase activity compared to diploid | Liu *et al*., 2011 |
| Drought | Withdrawal of watering of potted plants | *Citrus limonia* rootstocks grafted with *Citrus sinensis* scions | Spontaneous autotetraploid rootstocks vs. diploid | Increased drought-tolerance | Autotetraploid *C. limonia* seedlings show reduced stomatal density and stomatal conductance, increased expression of ABA synthesis and ABA responsive genes and of peroxidase 63 precursor.  When used as rootstocks 4x *C. limonia* showeds higher ABA content in both roots and leaves of *C. sinensis* scion. Leaves had lower stomatal conductance. | Allario *et al*., 2011;  Allario *et al*., 2013 |
| Drought | Polyethylene glycol treatment | *Nicotiana benthamiana* | Colchicine-induced octaploid vs. natural allotetraploid | Increased survival under drought stress | Octaploid showed delayed growth, larger stomata, lower stomatal density, higher antioxidant enzyme activity and concentration of ROS scavengers, and lower levels of lipid and protein damage than allotetraploid. | Deng *et al*., 2012 |
| Drought | Withdrawal of watering of potted plants | *Arachis duranensis* x *A. ipaënsis* synthetic allotetraploid | Colchicine-induced allotetraploids vs. diploid | Changes in some leaf traits favoured drought tolerance, while others do not. | Synthetic allotetraploid had thicker leaves, lower stomatal density on the adaxial surface and higher ratio of water storage cells layer/ leaf thickness than diploids, but its stomata remained open at lower soil water contents, showing a higher water loss per unit leaf area. | Leal-Bertioli *et al*., 2012 |
| Drought | Withdrawal of watering of potted plants | *Oryza sativa* | Colchicine-induced tetraploid vs. diploid | Increased drought-tolerance | Tetraploids retain more chlorophyll and photosynthetic activity, show lower levels of O_2_^-^ and lipid peroxidation, and higher activity of ROS detoxifying enzymes than diploids. | Yang P. M. *et al*., 2014 |
| Drought | Withdrawal of watering of potted plants | *Arabidopsis thaliana* Col-0 and Ler ecotypes | Synthetic autotetraploids vs. diploid | Increased drought-tolerance | Tetraploids have slower rates of whole-rosette water loss, enhanced stomatal closure, 3-fold lower stomatal density, decreased stomatal index and 20% larger stomatal pore. Tetraploids have constitutive ABA signaling and enhanced ROS production in leaves | Del Pozo and Ramirez-Parra, 2014 |
| Drought | Polyethylene glycol treatment | *Malus domestica* cv Hanfu and Gala | Colchicine-induced tetraploid vs. diploid | Increased drought-tolerance | Autotetraploids show delayed wilting, delayed symptoms of photooxidative stress and lowered expression of drought-inducible aquaporin genes | Zhang *et al*., 2015 |
| Drought | Reduced watering of potted plants | *Populus tremula* x *P. tremuloides* | Hybrid diploids vs. allotetraploids | Increased drought-tolerance | Tetraploids showed 60% longer stomata, 50% lower stomatal density, reduced water consumption, delayed wilting and more carbohydrate accumulation during drought than diploids | Hennig *et al*., 2015 |
| Drought | Potted plants subjected to different severities of drought by maintaining soil at 80, 50 or 20 % of maximum field  capacity | *Chamerion*  *angustifolium* | Naturally occurring diploid vs. hexaploid | Decreased drought-tolerance | Diploid shows higher photosynthetic and transpiration rates and higher water use efficiency than hexaploid under drought. Hexaploid shows Fv/Fm below normal under stress. Hexaploid has larger xylem conduits than diploid, suggesting higher susceptibility to cavitation. | Guo *et al*., 2016 |
| Drought | Withdrawal of watering of potted plants | *Lycium ruthenicum* | Colchicine-induced autotetraploid vs. diploid | Increased drought-tolerance | Up regulation of ABA biosynthesis and signal transduction pathways, increased accumulation of osmoprotectants. | Rao *et al*., 2020 |
| Drought | Potted plants subjected to soil water content of 4% | *Ziziphus jujuba* | Colchicine-induced autotetraploid vs. diploid | Increased drought-tolerance | Higher osmolyte content and antioxidant capacity in tetraploids under drought.  Tetraploids display a much larger number of differentially expressed genes under drought than diploids. Transcription factor expression enhanced in tetraploids. Authors attribute tolerance to gene duplication in tetraploid. | Li *et al*., 2021 |
| Drought | Potted young trees in greenhouse subjected to soil water content of 45% and 55% of pot capacity | Triploid *Citrus reticulata* × *Citrus sinensis*  and parental diploids | Hybridization | Increased drought tolerance | Triploid genotypes showed increased osmolyte content, stronger antioxidant defenses and enhanced photosynthetic capacity. | Lourkisti *et al*. 2022 |
| Drought | Potted plants subjected to moderate sustained drought (soil water potential = -30 kPa) | *Malus domestica cv. “M9”* grafted with diploid cv. ‘*Redchief*’ and  tetraploid clones | Synthetic autotetraploids vs. diploid | Increased drought tolerance | Tetraploids have thicker leaves with decreased stomatal density and larger stomata. Tetraploids maintain higher assimilation and transpiration rates under drought. Aquaporin gene expression is lowered and APX gene expression is enhanced in tetraploids under drought. | Wójcik *et al*., 2022 |

| ***Supplementary Table 2. Effect of polyploidy on responses to thermal stress. Within each type of thermal stress, entries were ordered according to the year of publication of the article referenced.*** | | | | | | |
| --- | --- | --- | --- | --- | --- | --- |
| **Stress** | **Experimental conditions** | ***Species*** | **Type of polyploidy** | **Polyploidization effect** | **Putative mechanisms involved** | **Reference** |
| Cold | crown-freezing technique. | *Lolium perenne* | Natural tetraploid vs. diploid | The 2x cultivars were more cold-tolerant than 4x. | No mechanism informed | Sugiyama *et al*., 1998 |
| Cold | Acclimation regime during three consecutive weeks at 5, 1 and -2 ºC each. | *Rhododendron cv. Rhododendron `PJM' and R. `Cunningham'sWhite'* | Diploid vs. tetraploid | The diploid  leaves hardened to -35.0ºC and the tetraploid ones to -22.7ºC. | No mechanism informed | Väinölä and Repo 1999 |
| Cold | Plants grown in field conditions from October 2003 to April 2004. Alt. 176 m a.s.l., long 16°48´E, lat 48°40´N | *Garden pansy (Viola × wittrockiana Gams)* | 8x, 10x, 12x, 14x, and 16x standard and hybrid cultivars | At –7.7°C, 16x plants were more sensitive, 12x intermediately sensitive, and 10x and 14x polyploids were as tolerant as the 8x standard controls. | No mechanism informed, though hexadecaploids showed a higher reduction of photosynthetic efficiency (Fv/Fm) at low temperatures than the remaining genotypes. | Lagibo *et al*., 2005 |
| Cold | Fresh leaves of rhizomes were exposed from −6 ◦C to −26 ◦C in 4◦C decrements, with each temperature maintained for 1 h. | *Dendranthema nankingense* | Colchicine-induced tetraploid vs. diploid | The 4x LT50 was 2.4 °C lower than the 2x | No mechanism informed | Liu *et al*., 2011 |
| Cold | Plants were grown under three temperature conditions: 25º (control), 10º and 4ºC for 7 days. | *Nicotiana benthamiana* | Colchicine-induced 4x and 8x | Survival time increased by 70% in 8x plants compared to 4x plants. | The activity of antioxidant enzymes (SOD, CAT, APX and GR) was greater in 4x than 8x. | Deng *et al*., 2012 |
| Cold | Excised leaves exposed to 0, -4, -8, -12, -16 and -20 °C for 12 h | *Strawberry cv. ‘Pink Princess and ‘YH15-10’* | Crosses and backcrosses.  between the pink-flowered strawberry cv. Pink Princess (8*x*) × synthetic white-flowered cv. YH15-10 (12*x*) | The cold tolerance of excised leaves from about 76.4 % of the analyzed hybrids was significantly superior to that of ‘Pink Princess’ leaves. | No mechanism informed | Xue L *et al*., 2015 |
| Heat | Leaves were treated for 2 h at 35 ◦C, then 2 h at 40 ◦C and stepwise in 5 ◦C increments in this fashion to 60 ◦C . | *Dendranthema nankingense* | Colchicine-induced tetraploid vs. diploid | The 2x LT50 was 1.2 ◦C greater than that of the 4x. | No mechanism informed | Li *et al*., 2009 |
| Heat | Seedlings of 2 months were treated with 38/23, 42/27, 45/30 or 48/33ºc day/night temperature regimes for 5d. | *Dioscorea zingiberensis* | Colchicine-induced tetraploid vs. diploid | Increased heat-tolerance | Tetraploid showed lower electrolyte leakage, products of lipid peroxidation and ROS content, as well as increased antioxidant metabolic markers than diploids | Zhang *et al*., 2010 |
| Heat | Ex-vitro grown plants exposed to a short term (0, 6 and 12 h) high temperature (32.5 °C) treatment | *Cnidium officinale* | Diploid vs. oryzalin- induced autotetraploid | Increased heat- tolerance | Tetraploid showed a lower increase of a heat-.stress induced cysteine protease (*CoCP*) than the diploid. Thermal resistance was associated to the increased content of phenolic compounds with antioxidant activity as a result of polyploidization | Kim *et al.*, 2021 |

***Supplementary Table 3. Effect of polyploidy on responses to saline stress. Entries were ordered according to the year of publication of the article referenced.***

| **Stress** | **Experimental conditions** | **Species** | **Type of polyploidy** | **Polyploidization effect** | **Putative processes involved** | **Reference** |
| --- | --- | --- | --- | --- | --- | --- |
| Salinity | Plants subjected for 28 days to 0, 100 and 200 mol m^-3^ NaCl in Hoagland nutrient solution | *Brassica sp.: B. napus, B. carinata, B. juncea, B. campestris, B. oleracea* and *B. nigra.* | Natural diploid vs tetraploid | Plant growth and seed yield was higher in amphiploids than diploids | Lower Na^+^ and higher K^+^ accumulation in tetraploid shoots and roots. | Ashraf *et al*., 2001 |
| Salinity | 250mM NaCl | *Isatis indigotica* | Natural autotetraploid vs. diploid | Increased tolerance to saline stress | Increment of expression of *liCPK2*, a saline stress response protein. | Lu *et al*., 2006 |
| Salinity | Irrigation with 50 mM NaCl (47 days) | *Citrus sinensis*  *Citrus reshni* *Poncirus trifoliata* | Natural autotetraploid vs. diploid. | Better growth rate and less chlorosis under saline stress | Lower Cl^-^  levels in the leaves | Saleh *et al*., 2008 |
| Salinity | Irrigation with 25,50 and 100 mM NaCl | *Trigonella foenum-graecum* | Colchicine-induced autotetraploid vs. diploid | Tetraploids showed enhanced tolerance to saline stress as evidenced by morphological traits evaluated | No mechanism informed | Marzougui *et al*., 2010 |
| Salinity | Irrigation with different concentrations of NaCl from 50 mM to 400 mM for 8 weeks. | *Citrus deliciosa* and *Poncirus trifoliata* | Natural autopolyploids and allotetraploid obtained with protoplast fusion vs. diploid | Increased sensitivity to saline stress | Cl^-^ accumulation in 4x leaves was greater and the maximum quantum yield of PSII was reduced in 4x as compared to 2x. | Mouhaya *et al*., 2010 |
| Salinity | Seeds germinated at 50, 100 and 200 mM NaCl during 7 days | *Brassica rapa* | Diploid vs. autotetraploid | Increased tolerance to salinity stress | Higher K^+^/Na^+^ ratio in roots, and higher glutathione levels and antioxidant activities in leaves of tetraploid than diploid genotypes. | Meng *et al*., 2011 |
| Salinity | Seedlings exposed to 200 mM NaCl for eight days. | *Dendranthema nankingense* | Colchicine-induced autotetraploid vs. diploid | Leaf chlorosis and necrosis were minor and delayed in tetraploids, which also showed a lower degree of lipid peroxidation than diploids | The SOD and POD activity remained higher and decreased more slowly in tetraploids. | Liu *et al*., 2011 |
| Salinity | Seeds germinated at different concentrations of NaCl (0-200 mM) | *Oryza sativa* | Colchicine-induced autotetraploid vs. diploid | In the absence of NaCl, tetraploids showed a lower germination rate than diploids. At increasing Na levels, seed mortality was lower, while seedling growth and chlorophyll a/b ratio was higher in the tetraploid as compared to the diploid | Free proline in tetraploid was greater. MDA content in tetraploid seedlings was lower, suggesting less oxidative damage. | Jiang *et al*., 2013 |
| Salinity | Irrigation with 200 mM NaCl | *Arabidopsis thaliana* | Natural and induced autotetraploids, and induced diploid. | Survival rate increase in tetraploid lines. | K^+^ increase and Na^+^ decrease in leaves. | Chao *et al*., 2013 |
| Salinity | Plants watered by soaking pots for 1 h twice a week in 50 mM NaCl for eight weeks. | *Citrus deliciosa* and *Citrus reshni* | Diploid vs. spontaneous autotetraploid | Increased tolerance to salinity stress | Higher constitutive levels of several ROS scavenging and photosynthesis-related enzymes in the 4x genotypes | Podda *et al*., 2013 |
| Salinity | Irrigation with 500 mM NaCl | *Robinia pseudoacacia* | Natural tetraploid vs. diploid | Tetraploids showed smaller negative effects than diploids in their morphological and physiological traits. | Increment in the levels of enzymes related to ROS scavenging and photosynthesis, energy proteins and defense proteins. | Wang *et al*., 2013 |
| Salinity | Seedlings  cultured in ½ MS medium  with 150 mM NaCl. | *Oryza sativa* | Colchicine-induced autotetraploid vs. diploid | Improved root tolerance to salt stress | Na^+^ concentration in tetraploid roots decreased while root tip H^+^ efflux significantly increased. | Tu *et al*., 2014 |
| Salinity | Seeds submerged in 10 mL of nutrient solution  supplemented with 100–500 mM of NaCl. Seedlings irrigated with 150 mM of NaCl for 32 or 36 d | *Triticum aestivum (6x)*  *Triticum turgidum (4x)*  Wheat genotype “Allo960”  *Aegilops tauschii (2x)* | Synthetic allohexaploid, natural allohexaploid and allotetraploid | Increment in seed germination and survival rate. | Stronger root Na^+^ retention capacity, and higher expression levels of the D-subgenome *HKT1;5* homeologue (*High-Affinity K+ Transporter*), which is responsible for Na^+^ removal from xylem vessels | Yang C *et al*., 2014 |
| Salinity | Irrigation with NaCl incremented 50 mM every day up to 300 mM for 15 days | *Lonicera japonica* | Colchicine-induced autotetraploid vs. diploid | Increased tolerance to saline stress | Lower Na^+^ accumulation in tetraploid leaves would help to maintain normal PSII and PSI coordination, thus preventing photoinhibition. | Yan *et al*., 2015 |
| Salinity | Seedlings treated with 200 mM^1^ NaCl for 8 days | *Malus domestica* | Colchicine-induced autotetraploid vs. diploid | Tetraploid maintained a higher relative water content (RWC) and lower proline and MDA levels than diploid. | Increased relative expression levels of aquaporin genes (MdPIP1;1, and MdTIP1;1) in the autotetraploid. | Xue H *et al*., 2015 |
| Salinity | Irrigation with 250 mM NaCl (15 and 30 days) | *Agave tequilana*  *Agave fourcroydes*  *Agave angustifolia* | Natural 2x (*A. tequilana and A. angustifolia*), 3x (*A. fourcroydes*), 5x (*A. fourcroydes*) and 6x allopolyploids (*A. angustifolia*) | Pentaploid and hexaploid more tolerant to saline stress than diploid and triploid | Increased expression of LEA genes | Tamayo-Ordóñez *et al*., 2016. |
| Salinity | Irrigation with 40 and 80 mM NaCl | *Citrus macrophylla* | Natural  autotetraploid vs. diploid | Moderate salinity reduced the biomass of 4x plants, but they had less damaged leaves under high salinity. | Lower Cl^-^  levels in the leaves | Ruiz *et al*., 2016 |
| Salinity | Plantlets cultured during 20 days in pots containing soil plus 0 (control), 0.2, 0.4, or 0.6 % NaCl (salt wt./ dry soil wt.) | *Paulownia fortunei* | Colchicine-induced autotetraploid vs. diploid | Enhanced tolerance to saline stress, as evidenced by higher relative water content, chlorophyll content, soluble sugar content, proline content, SOD activity, soluble protein content, and lower MDA levels. | 10 conserved and 10 novel miRNAs differentially expressed under salt treatment, among which eight were identified as miRNAs probably associated with higher salt tolerance. | Fan *et al*., 2016b |
| Salinity | Treatment with 70 mM NaCl for 0, 5, 10, 15, and 20 days for phenotyping. For physiological measurements: treatment with 0, 35, 70, 105 mM NaCl for 15 days. | *Paulownia tomentosa* | Colchicine-induced autotetraploid vs. diploid | Enhanced tolerance to saline stress | Changes in the expression of unigenes involved in salt stress response, assigned to several metabolic pathways, including “plant hormone signal transduction,” “RNA transporter,” “protein processing in endoplasmic reticulum,” and “plant-pathogen interaction,” | Fan *et al*., 2016a |
| Salinity | Irrigation with 0.4% NaCl every 3 days for 15 days | *Paulownia tomentosa* | Colchicine-induced autotetraploid vs. diploid | Enhanced tolerance to saline stress | Differences in the expression of genes functionally associated with plant hormone signal transduction, photosynthesis, and other metabolic pathways. | Zhao *et al*. 2017 |
| Salinity | Watered with salt solution (250 mM) for 2 weeks (once every 2 days) | *Hordeum bulbosum* | Natural tetraploid vs. diploid | Increased salinity tolerance as evidenced by reduced water loss in the leaves | Changes in the expression of five miRNAs whose targets are involved in protection of photosynthetic machinery against oxidative stress, osmotic stress-activated phospholipid signaling and salt stress response. | Liu and Sun, 2017 |
| Salinity | Seedlings treated with 0.2, 0.4, or 0.6% NaCl solution every 2 days for 15 days | *Paulownia australis* | Colchicine-induced autotetraploid vs. diploid | Morphological and physiological changes demonstrated that normal metabolism was less restrained in the tetraploid | Changes in the expression pattern of the common unigenes involved in soluble carbohydrate and proline metabolism, ROS-scavenging system, ion transporters, and transcription factors. | Dong *et al*., 2017 |
| Salinity | Treated with 0.4% NaCl solution every two days for 15 days | *Paulownia fortunei* | Colchicine-induced autotetraploid vs. diploid | Enhanced tolerance to saline stress | 152 differentially abundant proteins related to induced metabolism, signal transduction, or regulation of transcription. | Deng *et al*. 2017 |
| Salinity | Transplantation to NaCl/dry soil: 0, 0.2, 0.4,  and 0.6% (w:w) | *Paulownia fortunei* | Colchicine-induced autotetraploid vs. diploid | Enhanced tolerance to saline stress | Two dozens of genes were upregulated in tetraploids, 3 related to photosynthesis, 10 related to plant growth and development and 11 related to osmolytes (10 LEA proteins). | Wang *et al*., 2018 |
| Salinity | Seedlings exposed from 0 to 5 days to 50, 100, 200, and 300 mM NaCl | *Beta vulgaris* | Autotetraploid vs. diploid | Autotetraploid presented higher shoot biomass than diploid | Less accumulation of Na^+^ and soluble sugars, lower Na^+^/K^+^ ratio, greater K^+^- selective absorption capacity through the modulation of the expression levels of genes related to K^+^ and Na^+^ transport. | Wu *et al*., 2019 |
| Salinity | Subjected to 150 mM NaCl for 7 d. | *Ipomoea trifida* | Natural autohexaploid vs. diploid | Increased tolerance to salinity stress | Polyploids retained higher K^+^ and accumulated lower Na^+^ levels in root and leaves, apparently due to an increased sensitivity of root-zone-specific PM K^+^- and Ca^2+^-permeable channels to changes in H_2_O_2_ homeostasis. | Liu *et al*., 2019 |

***Supplementary Table 4. Effects of nutritional stress in polyploid plants. Entries were ordered according to the year of publication of the article referenced.***

| **Stress** | **Experimental conditions** | ***Species*** | **Type of polyploidy** | **Polyploidization effect** | **Putative processes involved** | **Reference** |
| --- | --- | --- | --- | --- | --- | --- |
| Boron deficiency | Seeds were sown on MGRL growth media containing 1% (w/v) sucrose. For preparation of low-B media, borate added to media was reduced. | *Arabidopsis thaliana* | Tetraploid lines obtained from Arabidopsis Biological Resource Center and diploid wild-types | Root cell elongation was improved in tetraploid under N deficiency, leading to improved root elongation of both the main and lateral roots. | A higher uptake surface might explain the enhanced tolerance to boron deficiency of tetraploids. | Kasajima *et al*., 2010 |
| Calcium Exces**s** | Plants were assigned to one of four treatment combinations (fully crossed fertilizer and calcium additions, each at a low and high level) and repotted in soil | *Solidago  gigantea* | Natural tetraploid and diploid from different locations. | Growth rate in tetraploids was less negatively affected than in diploids | No mechanism informed | Schlaepfer *et al*., 2010 |
| Boron Excess | Seedlings were grown for 45 days with 50 mM (control) or 400 mM H_3_BO_3_ (B treatment). | *Carrizo citrange (Citrus sinensis X L. Osb Poncirus trifoliata L. Raf.)* | Diploid and tetraploid genotypes from the Citrus Germplasm Bank. | 4x ploidy enhances B excess-tolerance. | Changes in root anatomical features of 4x plants would play a key role in lowering B uptake capacity and root-to-shoot B transport. | Ruiz *et al*., 2016 |
| Nitrogen deficiency | The pots were watered with an altered half-strength Hoagland nutrient solution (0.1 mMNO3-, pH 6.5) for 7 or 31 days. | *Triticum turgidum* | Natural tetraploid, diploid and allohexaploid. | At low N levels, 6x maintained largely normal photosynthesis, higher shoot N accumulation, and better N assimilation than its 4x and 2x parents. | Hexploids showed higher root/shoot ratio and H^+^ efflux, and displayed higher expression levels of critical genes involved in N uptake than both of its 4x and 2x parents. | Yang *et al*.,2018 |

***Supplementary Table 5. Effect of biotic stress in polyploid plants. Data were ordered according to the year of publication in the reference list***

| **Stress** | **Experimental conditions** | **Species** | **Type of polyploidy** | **Polyploidization effect** | **Putative processes involved** | **Reference** |
| --- | --- | --- | --- | --- | --- | --- |
| Biotic | Adult chinch bugs confined for 7 day with grass stolon terminals in plastic bags | *Stenotaphrum secundatum*  *S. dimidiatum* | Diploid vs tetraploid | Higher levels of resistance (antibiosis) in some polyploid cultivars | No mechanism informed | Reinert *et al*., 1986 |
| Biotic | Seedlings inoculated by dusting leaves with dry spores of *Phakopsora pachyrhizi* born on infected soybean plants. Inoculated seedlings were then sprayed with a fine mist of water and stored overnight at 100% relative humidity before being placed in a naturally lit glasshouse | *Glycine tomentella* | Natural allotetraploid vs. diploid | Similar levels of resistance between polyploids and diploids | No mechanism informed | Schoen et al., 1992 |
| Biotic | Plants inoculated with *Belonolaimus longicaudatus* per pot, pipetted  in 2 mL water into a 10-mm-deep soil depression near the  most proximal rooted node. | *Stenotaphrum secundatum* | Natural autotetraploid vs. diploid | Polyploids supported lower nematode numbers and plant performance defects than diploids. | Polyploid *Stenotaphrum* genotypes tend to have thicker primary roots than diploids. | Busey *et al*., 1993 |
| Biotic | Plants and adult moth *Greya politella* surveys (plant attack) | *Heuchera grossulariifolia* | Natural autotetraploid vs. diploid | Tetraploid plants more susceptible to attack from moths than diploids | In some regions tetraploid plants generally flower earlier than diploid plants. As a result, flowering of tetraploids partially overlaps the flowering of an alternative host species of *G. politella*, whereas flowering of diploids generally does not. | Thompson *et al*., 1997 |

References:

Hennig, A., Kleinschmit, J. R., Schoneberg, S., Löffler, S., Janßen, A., and Polle, A. (2015). Water consumption and biomass production of protoplast fusion lines of poplar hybrids under drought stress. Front. Plant Sci. 6, 330. doi: 10.3389/fpls.2015.00330

Leal-Bertioli, S. C., Bertioli, D. J., Guimarães, P. M., Pereira, T. D., Galhardo, I., Silva, J. P., et al. (2012). The effect of tetraploidization of wild Arachis on leaf morphology and other drought-related traits. Environ. Exp. Bot. 84, 17–24. doi: 10.1016/j.envexpbot.2012.04.005

Liu, Y., Yu, Y., Sun, J., Cao, Q., Tang, Z., Liu, M., et al. (2019). Root-zone-specific sensitivity of K+ and Ca2+ permeable channels to H2O2 determines ion homeostasis in salinized diploid and hexaploidy Ipomoea trifida. J. Exp. Bot. 70, 1389–1405. doi: 10.1093/jxb/ery461

Marzougui, N., Guasmi, F., Elfalleh, W., Boubaya, A., Touil, L., Ferchichi, A., et al. (2010). Physiological performance of an induced autotetraploid genotype of Trigonella foenum-graecum L. compared with diploid genotypes. Acta Botanica Gallica. 157, 117–126. doi: 10.1080/12538078.2010.10516193

Mouhaya, W., Allario, T., Brumos, J., Andres, F., Froelicher, Y., Luro, F., et al. (2010). Sensitivity to high salinity in tetraploid citrus seedlings increases with water availability and correlates with expression of candidate genes. Funct. Plant Biol. 37, 674–685. doi: 10.1071/FP10035

Reinert, J. A., Busey, P., and Bilz, F. G. (1986). Old world St. Augustinegrasses resistant to the southern chinch bug (Heteroptera: Lygaeidae). J. Econ. Entomol. 79, 1073–1075. doi: 10.1093/jee/79.4.1073

Schoen, D. J., Burdon, J. J., and Brown, A. H. D. (1992). Resistance of Glycine tomentella to soybean leaf rust Phakopsora pachyrhizi in relation to ploidy level and geographic distribution. Theoret. Appl Genetics. 83, 827–832. doi: 10.1007/BF00226704

Väinölä, A., and Repo, T. (1999). Cold hardiness of diploid and corresponding autotetraploid rhododendrons. J. Hortic. Sci. Biotechnol. 74, 541–546. doi: 10.1080/14620316.1999.11511149

Wu, G. Q., Lin, L. Y., Jiao, Q., and Li, S. J. (2019). Tetraploid exhibits more tolerant to salinity than diploid in sugar beet (Beta vulgaris L.). Acta Physiol. Plant. 41, 1–17. doi: 10.1007/s11738-019-2844

Yan, K., Wu, C., Zhang, L., and Chen, X. (2015). Contrasting photosynthesis and photoinhibition in tetraploid and its autodiploid honeysuckle (Lonicera japonica Thunb.) under salt stress. Front. Plant Sci. 6, 227. doi: 10.3389/fpls.2015.00227
